# Usefulness of grayscale values of hypoechoic lesions matched with target lesions observed on magnetic resonance imaging for the prediction of clinically significant prostate cancer

**DOI:** 10.1186/s12894-022-01111-7

**Published:** 2022-10-29

**Authors:** Dong Gyun Kim, Jeong Woo Yoo, Kyo Chul Koo, Byung Ha Chung, Kwang Suk Lee

**Affiliations:** 1 Department of Urology, National Health Insurance Service Ilsan Hospital, Goyang, Republic of Korea; 2 Department of Urology, Gangnam Severance Hospital, Urological Science Institute, Yonsei University College of Medicine, 211 Eonju-ro, Gangnam-gu, Seoul, 06273 Republic of Korea

**Keywords:** Grayscale, Hypoechoic lesion, Prostate imaging-reporting and data system, Prostate cancer, Target biopsy

## Abstract

**Background:**

To analyze grayscale values for hypoechoic lesions matched with target lesions evaluated using prebiopsy magnetic resonance imaging (MRI) according to the Prostate Imaging-Reporting and Data System (PI-RADS).

**Methods:**

We collected data on 420 target lesions in patients who underwent MRI/transrectal ultrasound fusion-targeted biopsies between January 2017 and September 2020. Images of hypoechoic lesions that matched the target lesions on MRI were stored in a picture archiving and communication system, and their grayscale values were estimated using the red/green/blue scoring method through an embedded function. We analyzed imaging data using grayscale values.

**Results:**

Of the 420 lesions, 261 (62.1%) were prostate cancer lesions. There was no difference in the median grayscale values between benign and prostate cancer lesions. However, grayscale ranges (41.8–98.5 and 42.6–91.8) were significant predictors of prostate cancer and clinically significant prostate cancer (csPC) in multivariable logistic regression analyses. Area under the curve for detecting csPC using grayscale values along with conventional variables (age, prostate-specific antigen levels, prostate volume, previous prostate biopsy results, and PI-RADS scores) was 0.839, which was significantly higher than that for detecting csPC using only conventional variables (0.828; *P* = *0.036*). Subgroup analysis revealed a significant difference for PI-RADS 3 lesions between grayscale values for benign and cancerous lesions (74.5 vs. 58.8, *P* = *0.008*). Grayscale values were the only significant predictive factor (odds ratio = 4.46, *P* = *0.005)* for csPC.

**Conclusions:**

Distribution of grayscale values according to PI-RAD 3 scores was potentially useful, and the grayscale range (42.6–91.8) was a potential predictor for csPC diagnosis.

## Background

Prebiopsy magnetic resonance imaging (MRI) is recommended for patients suspected of having prostate cancer (PCa) in the screening test [1]. Results of prostate MRI are classified based on the presence or absence of visible lesions. For patients with visible lesions, the likelihood of PCa diagnosis increases with the detection of lesions [2]. Introduction of the Prostate Imaging-Reporting and Data System (PI-RADS) for the diagnosis of clinically significant PCa (csPC) standardized the interpretation of MRI results for visible lesions [3]. The rate of PCa diagnosis increases with increasing PI-RADS scores (PI-RADS 3: 29.7%, PI-RADS 4: 42.3%, PI-RADS 5: 82.4%) [4]. For improving cost-effectiveness, new strategies for improving the diagnostic accuracy of PI-RADS have been investigated [5–7]. For improving the specificity of screening, the National Comprehensive Cancer Network Clinical Practice Guidelines in Oncology for PCa Early Detection recommend using tests and scores associated with biomarkers levels, such as the Prostate Health Index, SelectMDx® test, 4Kscore® test, and ExoDx™ Prostate Test, as additional useful tools [8]; however, biomarkers were not considered the diagnostic tool for evaluating target lesions on MRI.

Van de Ven et al. reported that the presence of a clearly hypoechoic lesion in MRI/transrectal ultrasound (TRUS) fusion-targeted biopsy was associated with the prediction of high-grade PCa [9]. Therefore, we hypothesized that analysis of target lesions on TRUS matched with those observed on MRI during MRI/TRUS fusion-targeted biopsies might be useful for stratifying the lesions according to the PI-RADS scores. In addition, we conducted an experimental study to evaluate whether, with respect to systematic biopsies, high-grade PCa lesions can be subdivided if grayscale values for hypoechoic lesions are considered in addition to conventional variables such as age, prostate-specific antigen (PSA) levels, and prostate volume (PV) [10]. This study aimed to confirm the effectiveness of analyzing hypoechoic lesions using grayscale values. Additionally, we suggested a range of grayscale values for improving the prediction of PCa in addition to the PI-RADS.

## Methods

### Patient selection

This study was approved by the institutional ethics committee (Yonsei University Health System, Seoul, Korea, 3-2020-0236), and all procedures were conducted in accordance with the ethical standards of the 1964 Helsinki declaration and its later amendments. The requirement for informed consent was waived for this study by Yonsei University Health System as it was based on retrospective, anonymous patient data and did not involve patient intervention or the use of human tissue samples.

We collected data on 449 consecutive patients who underwent MRI/TRUS fusion-targeted biopsies at our institution between January 2017 and September 2020. We recommended prostate biopsy for patients with PSA levels > 3.0 ng/mL with palpable nodules identified through digital rectal examinations, with or without continuous elevations in PSA levels during follow-up periods, with or without relevant prebiopsy MRI findings.

Of the 580 target lesions in the 449 patients (median age: 69.3 years [interquartile range: 62.9–75.3]; median PSA level: 7.02 ng/mL [5.00–11.00]; median PV: 32.8 cm^3^ [25.7–45.9], median PSA density (PSA level divided by PV): 0.20 ng/mL/cm^3^ [0.13–0.36]; number of patients diagnosed with PCa: 312 [69.5%]; number of patients with previous prostate biopsy history: 100 [22.3%]; cancer detection rate [CDR] in patients with PI-RADS 3 lesions: 57/180 [31.7%]; CDR in patients with PI-RADS 4 lesions: 156/255 [61.2%]; CDR in patients with PI-RADS 5 lesions: 136/150, [90.7%]), 160 target lesions were excluded from the study for the following reasons: (i) for 120 patients, no TRUS images were obtained during MRI/TRUS fusion-targeted biopsies; (ii) for 16 patients, the quality of ultrasound images was poor; and (iii) three patients were diagnosed as having neuroendocrine tumors. Finally, 420 target lesions were included in this study.

### Data collection

Data on patient characteristics, including clinicopathological data such as age, history of prostate biopsy, PV, PSA level, PSA density, MRI findings, and TRUS images, were obtained.

### MRI protocol and imaging analysis

Imaging was performed using a 3-T MRI system (Intera Achieva 3.0 T, Phillips Medical System, Best, the Netherlands) equipped with a six-channel phased-array coil. The prostate MRI protocol involved diffusion-weighted imaging in addition to T2-weighted imaging. Images were acquired through T2-weighted turbo spin-echo MRI in three planes (axial, sagittal, and coronal). MRI datasets were obtained at identical slice locations, with a slice thickness of 3 mm and no intersection gap. B-values (range: 0–1400 s/mm^2^) were used, and diffusion restriction was quantified via apparent diffusion coefficient mapping. Dynamic contrast-enhanced MRI was also performed.

Uro-radiologists denoted suspicious regions of interest on the apparent diffusion coefficient maps examined using the Digital Imaging and Communications in Medicine workstation. The PI-RADS scoring system was used to describe MRI findings [3]. Visible lesions were defined as lesions with PI-RADS scores ≥ 3.

### MRI-targeted biopsy technique

Prostate biopsies were performed after periprostatic nerve blocks had been administered using Chiba needles. Initially, four MRI-targeted cores biopsies for each targeted lesion were performed, followed by 12-core biopsies. MRI/TRUS fusion targeted biopsies were performed with an MRI/TRUS-fusion-targeted-biopsy protocol using the bk3000 ultrasound system, which involves the use of a side-fire ultrasound probe (BK Medical, Peabody, MA, USA) and an image-based fusion system (BioJet; GeoScan, Lakewood Ranch, FL, USA). All prostate biopsies were performed by an experienced urologist using an 18-gauge, 20-cm disposable core biopsy instrument (Max-Core™; BD, BD Headquarters, NJ, USA).

### Ultrasound image analysis

Ultrasound images of target lesions observed on MRI were stored concomitantly using a picture archiving and communication system (PACS) (GE Healthcare, Barrington, IL, USA). We analyzed the grayscale version of the images using a red/green/blue (RGB) scoring method through a function embedded in the PACS. An average RGB value was obtained from scores at three other randomized points in the most identical slice. Grayscale values were replaced with RGB values on a pixel-by-pixel basis (*Y* = 0.2126 * *R* + 0.7152 * *G* + 0.0722 * *B*) [11]. Two investigators measured the average of three points, and we confirmed there were no differences in grayscale through paired sample t-tests.

### Study objectives

Our primary objective was to investigate the efficacy of imaging analysis by quantifying hypoechoic lesions in MRI-target lesions, demonstrating its diagnostic accuracy, and specifying the quantitation range of hypoechoic lesions for the prediction of PCa and csPC. Our secondary objective was to identify the grayscale range for improving the prediction of PCa and csPC in addition to PI-RADS.

### Statistical analysis

Continuous variables are expressed as medians (interquartile range) or mean ± standard deviation, and categorical variables as number of occurrences and frequency. Pearson’s χ2 test was used for statistical comparisons of continuous and categorical variables. Univariable and multivariable logistic regressions were used. Receiver operating characteristic (ROC) curves and areas under the ROC curves (AUCs) were used to evaluate the performance of standard clinical parameters (including age, previous prostate biopsy history, PSA level, PV, and PI-RADS score) versus the grayscale values in addition to standard clinical parameters for the diagnosis for PCa and csPC (defined as PCa of Gleason grade group ≥ 2) for target lesions. Pairwise comparisons of ROC curves were conducted to compare the predictive performance of individual and combined parameters. The cut-off value was assessed from the AUCs. These optimal cut-off values were based on predefined values and an analysis performed using the Youden index (sensitivity + specificity − 1). All statistical comparisons were conducted with IBM® SPSS® Statistics 25 (IBM, Armonk, NY, USA) and MedCalc version 11.6 (MedCalc Software Ltd, Acacialaan, Ostend, Belgium). For differences, *P* < *0.05* was considered statistically significant.

## Results

### Baseline characteristics of the patients and grayscale

Baseline characteristics of the patients and target lesions are shown in Table 1. Age, PSA level, PV, and the PI-RADS score were significantly different between the benign and PCa groups. The CDR was 36.1% (43/119) in patients with PI-RADS 3 lesions, 62.7% (126/201) in patients with PI-RADS 4 lesions, and 92.0% (92/100) in patients with PI-RADS 5 lesions (Table 1). There were no significant differences between the grayscale values for benign and cancerous lesions (70.3 vs. 65.9, *P* = *0.359*). The grayscale range for predicting PCa was 41.8–98.5 and that for predicting csPC was 42.6–91.8.

**Table 1:** Characteristics of patients and target lesions

	Prostate cancer		
	No (n = 159)	Yes (n = 261)	*P* value
Age (years)	66.7 ± 8.5	70.4 ± 7.8	< *0.001*
PSA (ng/mL)	5.83 (4.60–8.09)	7.97 (5.37–14.22)	*0.001*
Prostate volume (cm^3^)	41.2 ± 15.2	34.0 ± 15.1	< *0.001*
PSAD (ng/mL/cm^3^)	0.15 (0.11–0.23)	0.27 (0.17–0.47)	*0.002*
Previous prostate biopsy history	43 (27.0)	41 (15.7)	*0.007*
Positive systematic biopsy	35 (22.0)	233 (89.3)	< *0.001*
Gleason grade group			< *0.001*
1	17 (48.6)	59 (25.3)	
≥ 2	18 (51.4)	174 (74.7)	
No. of positive systematic biopsy cores	0.6 ± 1.4	4.6 ± 3.4	< *0.001*
Target-lesion location			*0.642*
Peripheral zone	37 (23.3)	66 (25.3)	
Transitional zone	122 (76.7)	195 (74.7)	
Target-lesion size (cm)	1.01 ± 0.80	1.59 ± 1.09	< *0.001*
PI-RADS score			< *0.001*
3	76 (63.9)	43 (36.1)	
4	75 (37.3)	126 (62.7)	
5	8 (8.0)	92 (92.0)	
Grayscale value	70.3 (45.3–98.4)	65.9 (47.7–89.9)	*0.359*

Data are expressed as number (%) and median (interquartile range)

*PI-RADS* prostate imaging-reporting and data system, *PSA* prostate-specific antigen, *PSAD* prostate-specific antigen density

### Grayscale values as a predictor of PCa and csPC

Univariable analysis for PCa and csPC identified age, PSA level, PV, previous prostate biopsy history, the PI-RADS score, and grayscale values as potential predictors of PCa and csPC (Table 2). Multivariable analysis to evaluate the predictive ability of grayscale values in addition to conventional variables revealed that grayscale values were a significant predictor of PCa and csPC (Table 3).

**Table 2:** Logistic regression analyses for the prediction of prostate cancer

	Univariable analysis		Multivariable analysis	
	OR (95% CI)	*P* value	OR (95% CI)	*P* value
Age	1.06 (1.032–1.085)	< *0.001*	1.05 (1.020–1.086)	*0.001*
PSA	1.06 (1.028–1.101)	< *0.001*	1.05 (1.006–1.092)	*0.025*
Prostate volume	0.97 (0.957–0.983)	< *0.001*	0.96 (0.942–0.975)	< *0.001*
Previous prostate biopsy history	0.50 (0.310–0.815)	*0.049*	0.57 (0.322–0.999)	*0.050*
PI-RADS		< *0.001*		< *0.001*
4 vs. 3	2.97 (1.854–4.754)	< *0.001*	2.62 (1.544–4.449)	< *0.001*
5 vs. 3	20.33 (9.010–45.851)	< *0.001*	12.70 (5.268–30.597)	< *0.001*
Grayscale values (41.8–98.5)	1.63 (1.088–2.432)	*0.018*	1.77 (1.087–2.868)	*0.022*

*CI* confidence interval, *OR* odds ratio, *PI-RADS* prostate imaging-reporting and data system, *PSA* prostate-specific antigen

**Table 3:** Logistic regression analyses for the prediction of clinically significant prostate cancer

	Univariable analysis		Multivariable analysis	
	OR (95% CI)	*P* value	OR (95% CI)	*P* value
Age	1.06 (1.032–1.085)	< *0.001*	1.05 (1.018–1.085)	*0.002*
PSA level	1.05 (1.027–1.082)	< *0.001*	1.04 (1.007–1.064)	*0.014*
Prostate volume	0.97 (0.959–0.986)	< *0.001*	0.96 (0.938–0.973)	< *0.001*
Previous prostate biopsy history	0.55 (0.332–0.899)	*0.017*	0.71 (0.390–1.294)	*0.264*
PI-RADS score		< *0.001*		< *0.001*
4 vs. 3	4.01 (2.304–6.992)	< *0.001*	3.88 (2.104–7.163)	< *0.001*
5 vs. 3	25.99 (12.664–53.326)	< *0.001*	20.375 (9.211–45.069)	< *0.001*
Grayscale values (42.6–91.8)	1.72 (1.162–2.537)	*0.007*	2.11 (1.298–3.443)	*0.003*

*CI* confidence interval, *OR* odds ratio, *PI-RADS* prostate imaging-reporting and data system, *PSA* prostate-specific antigen

**Fig. 1:**
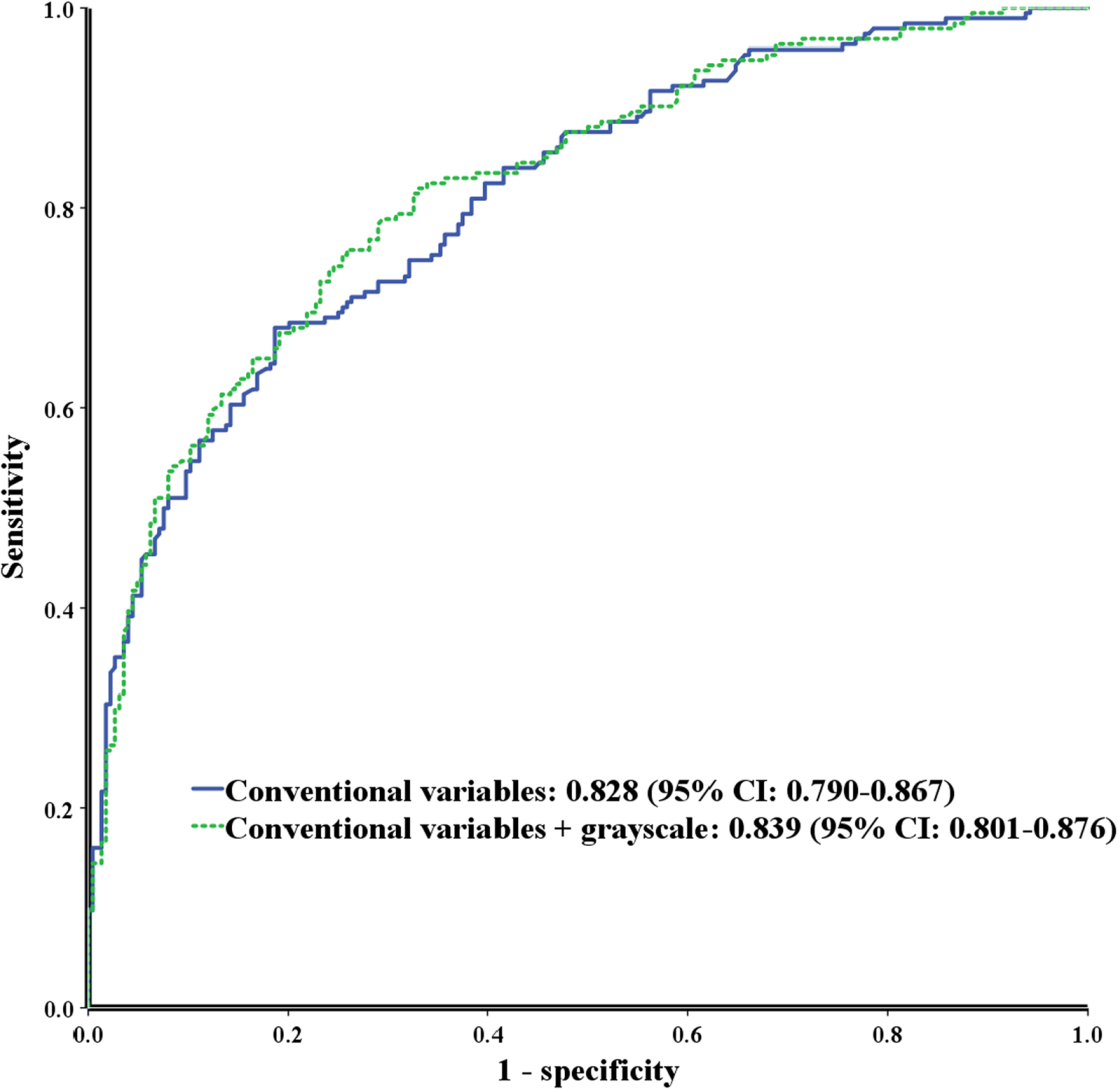
The ROC curves for detecting csPC using conventional variables versus grayscale values in addition to conventional variables ROC. ROC, receiver operating characteristic; csPC. Clinically significant prostate cancer

The AUC for detecting csPC using grayscale values in addition to conventional variables was 0.839, which was significantly higher than that for detecting csPC using only conventional variables (0.828; *P* = *0.036*) (Fig. 1). The sensitivity and specificity of grayscale values were 62.4% and 50.9%, respectively. However, regarding predicting PCa, no differences were found between prediction using only conventional variables and that using grayscale values in addition to conventional variables (0.828 vs. 0.817, *P* = *0.145*).

### Usefulness of grayscale values for predicting csPC in patients with PI-RADS 3 lesions

We analyzed the prediction of PCa and csPC using the grayscale values for all PI-RADS scores (Table 4). For groups of individuals with cancer of the same PI-RADS score, the proportion of individuals with high-grade cancer increases with increasing PI-RADS scores (*P* < *0.001*). For cancer lesions, grayscale values showed the tendency of increasing and paradoxically decrease with increasing PI-RADS scores (PI-RADS 3: 58.8 [44.8–94.3], PI-RADS4: 73.2 [50.0–93.7], PI-RADS 5: 65.6 [47.7–92.2]). Regarding PI-RADS 3 lesions, the median grayscale values were significantly different between cancerous and benign lesions (58.8 [44.8–94.3] and 74.5 [54.8–99.2], respectively; *P* = *0.008*), whereas for lesions with PI-RADS score of 4 or 5, there was no significant difference between the grayscale values.

**Table 4:** Gleason grade groups and grayscale values according to the PI-RADS scores of target lesions

	PI-RADS 3	PI-RADS 4	PI-RADS 5	*P* value
Gleason grade group				< *0.001*
1	23 (53.5)	36 (28.6)	8 (8.7)	
2–3	12 (27.9)	64 (50.8)	41 (44.6)	
4–5	8 (18.6)	26 (20.6)	43 (46.7)	
Grayscale values				
Total	65.6 (47.0–94.7)	69.7 (45.5–93.7)	65.7 (47.7–91.7)	*0.915*
Benign	74.5 (54.8–99.2)	61.5 (39.5–93.7)	82.9 (34.5–91.5)	*0.304*
Cancerous	58.8 (44.8–94.3)	73.2 (50.0–93.7)	65.6 (47.7–92.2)	*0.077*
*P* value	*0.008*	*0.398*	*0.838*	

Data are expressed as number (%) and median (interquartile range)

*PI-RADS* prostate imaging-reporting and data system

For PI-RADS 3 lesions, the grayscale cut-off range for predicting PCa was 36.2–89.7 and that for predicting csPC was 42.9–71.9 (Table 5). The grayscale cut-off value of prostate cancer was 61.3 and that of clinically significant prostate cancer was 58.4. The multivariable analysis in which variables such as age, PV, and grayscale values were considered identified age (odds ratio [OR] = 1.08; confidence interval [CI]: 1.026–1.134; *P* = *0.003*) and PV (OR = 0.95; CI 0.911–0.980; *P* = *0.002*) as predictors of PCa diagnosis. Grayscale values were not found to be a significant predictor. The AUC for detecting PCa using grayscale values in addition to conventional variables was 0.702, which was significantly higher than that for detecting PCa using only conventional variables (0.656; *P* = *0.001*). For csPC, grayscale values were identified as the only independent significant predictor (OR = 4.46; CI 1.573–12.652; *P* = *0.005*). For PI-RADS 3 lesions, the sensitivity and specificity of the grayscale range (42.9–71.9) for detecting csPC were 0.487 and 0.767, respectively.

**Table 5:** Logistic regression analyses for predicting the presence of prostate cancer and clinically significant prostate cancer in PI-RADS 3 lesion

	Univariable analysis		Multivariable analysis	
	OR (95% CI)	*P* value	OR (95% CI)	*P* value
Cancer				
Age	1.07 (1.017–1.116)	*0.007*	1.08 (1.026–1.134)	*0.003*
PSA level	1.02 (0.980–1.060)	*0.346*		
Prostate volume	0.95 (0.919–0.983)	*0.003*	0.95 (0.911–0.980)	*0.002*
Previous prostate biopsy history	0.91 (0.378–2.186)	*0.831*		
Grayscale values (36.2–89.7)	3.13 (1.354–7.240)	*0.008*	2.26 (0.905–5.624)	*0.081*
Clinically significant prostate cancer				
Age	1.04 (0.985–1.106)	*0.146*		
PSA	1.01 (0.966–1.053)	*0.691*		
Prostate volume	0.96 (0.919–1.0020)	*0.062*		
Previous prostate biopsy history	0.74 (0.226–2.422)	*0.619*		
Grayscale values (42.9–71.9)	4.46 (1.573–12.652)	*0.005*		

*CI* confidence interval, *OR* odds ratio, *PI-RADS* prostate imaging-reporting and data system, *PSA* prostate-specific antigen

## Discussion

The presence of visible lesions on prebiopsy MRI increases the likelihood of PCa diagnosis. To classify visible lesions identified on prebiopsy MRI, the PI-RADS was developed for predicting csPC and interpreting MRI results [3, 12]. In order to increase the efficacy of using prebiopsy MRI results for the prediction of PCa and csPS [13–16], new methods for the classification of lesions are needed. We hypothesized that image analysis of hypoechoic lesions on TRUS matched with target lesions on MRI in MRI/TRUS fusion-targeted biopsies could be used effectively for predicting PCa and csPC. Using grayscale values in addition to conventional variables was helpful for increasing the probability of correctly detecting csPC. Especially, regarding PI-RADS 3 lesions, grayscale values were identified as the only discriminating factor for benign and cancerous lesions.

Until now, several studies have reported that the presence of hypoechoic lesions with definite visibility in TRUS is related to the diagnosis of csPC with or without visible lesions on MRI [2, 17]. However, in several guidelines, hypoechoic lesions have not been included as indicators for prostate biopsy [1, 8, 18]. The reasons were considered as lack of reproducibility and representativeness associated with subjective measurements of hypoechoicity for selecting hypoechoic lesions and the fact that increasing cancer aggressiveness has an effect on fibrotic change and echogenicity, which show apparent differences on ultrasound images of PCa [19]. To overcome limitations regarding reproducibility and representativeness, in our previous study, we reported the usefulness of grayscale values for the quantitation and stratification of hypoechoic lesions on cancer aggressiveness [10]. In the present study, through quantification with grayscale values, we showed that the characteristics of visible lesions observed through TRUS reaffirmed the results of a previous study [2].

To the best of our knowledge, no studies involving the use of numerical analyses, such as the analysis of grayscale values for explaining the relationship between increasing cancer aggressiveness and hypoechoicity, have been conducted. Therefore, we collected and analyzed the images obtained during MRI/TRUS fusion-targeted biopsies. Regarding the measurement of grayscale values for target lesions, no difference was found between the median grayscale values for benign and cancerous lesions. However, the grayscale value interquartile range for benign lesions was larger than that for cancerous lesions. Therefore, we identified the optimal grayscale range for predicting PCa and csPC. Finally, we confirmed that the grayscale range was a significant predictive factor of PCa and csPC. A combined evaluation of clinical variables and images increases the accuracy of predicting csPC, which is consistent with the results of our previous study [10].

For PI-RADS 3 lesions, there was a relatively low predicting ability for PCa diagnosis [20–22]. Therefore, several researchers investigated the discrimination of PI-RADS 3 lesions using clinical parameters and biomarkers [23, 24]. Sheridan et al. reported that when PI-RADS 3 lesions are present, csPC detection rates can be increased by evaluating factors such as clinical parameters (age ≥ 70 years, size ≤ 36 cm^3^, positive digital rectal examination) [7]. Another study found that evaluation of the Prostate Health Index in addition to MRI results improved the predictive performance for overall cancer (AUC 0.71 vs. 0.60) and csPC detection (AUC 0.75 vs. 0.64) [5]. Our findings may improve the rate of accurate overall cancer diagnosis (AUC 0.70 vs. 0.66) among individuals with PI-RADS 3 lesions. To predict csPC in PI-RAD 3, the grayscale characteristics of target lesions were the only factor in this study.

Certain meaningful findings were made in this study. First, we presented the grayscale range for predicting csPC through a quantitative image analysis of target lesions matched in MRI/TRUS fusion-targeted biopsies. This use of grayscale values is a novel method to stratify suspicious cancer lesions on MRI. Second, previous studies on PCa diagnosis in hypoechoic lesions estimated with low accuracy due to several reasons. Through this study, we present objective findings that explain correlations between echogenicity and PI-RADS, which were not made in previous studies [19]. Measurement of the grayscale values requires minimal expenditure because they are measured using ultrasound, which is used for TRUS, and PACS can be used to store TRUS results, instead of a new equipment. A limitation of our study is that it was a single-institution study. In particular, the number of PI-RADS 3 lesions was small, resulting in paradoxical results using logistic regression analysis and pairwise comparison of ROCs for prostate cancer diagnosis in a subgroup analysis of PI-RADS 3 lesions. Therefore, for external validation, a multicenter study should be performed in the future. Moreover, it is necessary to study whether different results would be obtained with ultrasonic equipment produced by different companies.

## Conclusions

The efficacy of prebiopsy MRI has been proven; however, regarding PCa detection, results associated with PI-RADS 3 lesions have been equivocal. We confirmed the distribution of grayscale values according to the PI-RADS scores and found that the grayscale values of hypoechoic lesions on TRUS are potential predictor of csPC. Our results showed that for PI-RADS 3 lesions, grayscale values were potentially useful to predict csPC, and the cut-off range was 42.9–71.9.

## Data Availability

The datasets used and analyzed during the current study are available from the corresponding author on reasonable request.

## Abbreviations

AUC: Area under the receiver operating characteristic curve
CDR: Cancer detection rate
csPC: Clinically significant prostate cancer
MRI: Magnetic resonance imaging
PACS: Picture archiving and communication system
PCa: Prostate cancer
PI-RADS: Prostate imaging-reporting and data system
PSA: Prostate-specific antigen
PV: Prostate volume
RGB: Red/green/blue
ROC: Receiver operating characteristic
TRUS: Transrectal ultrasound

## References

[1] Carroll PR, Parsons JK, Carlsson S (2020). NCCN guidelines insights: prostate cancer early detection, version 2.2020. J Natl Compr Canc Netw.

[2] Choi MH, Lee YJ, Jung SE, Lee JY, Choi YJ (2019). Prostate cancer detection rate according to lesion visibility using ultrasound and MRI. Clin Radiol.

[3] Kasel-Seibert M, Lehmann T, Aschenbach R, Guettler FV, Abubrig M, Grimm MO, Teichgraeber U, Franiel T (2016). Assessment of PI-RADS v2 for the detection of prostate cancer. Eur J Radiol.

[4] Sathianathen NJ, Konety BR, Soubra A, Metzger GJ, Spilseth B, Murugan P, Weight CJ, Ordonez MA, Warlick CA (2018). Which scores need a core? An evaluation of MR-targeted biopsy yield by PIRADS score across different biopsy indications. Prostate Cancer Prostatic Dis.

[5] Gnanapragasam VJ, Burling K, George A, Stearn S, Warren A, Barrett T, Koo B, Gallagher FA, Doble A, Kastner C (2016). The Prostate Health Index adds predictive value to multi-parametric MRI in detecting significant prostate cancers in a repeat biopsy population. Sci Rep.

[6] Fan YH, Pan PH, Cheng WM, Wang HK, Shen SH, Liu HT, Cheng HM, Chen WR, Huang TH, Wei TC (2021). The Prostate Health Index aids multi-parametric MRI in diagnosing significant prostate cancer. Sci Rep.

[7] Sheridan AD, Nath SK, Syed JS, Aneja S, Sprenkle PC, Weinreb JC, Spektor M (2018). Risk of clinically significant prostate cancer associated with prostate imaging reporting and data system category 3 (Equivocal) lesions identified on multiparametric prostate MRI. AJR Am J Roentgenol.

[8] Carroll PR, Parsons JK, Andriole G, Bahnson RR, Castle EP, Catalona WJ, Dahl DM, Davis JW, Epstein JI, Etzioni RB (2016). NCCN guidelines insights: prostate cancer early detection, version 2.2016. J Natl Compr Canc Netw.

[9] van de Ven WJ, Sedelaar JP, van der Leest MM, van de Hulsbergen KCA, Barentsz JO, Futterer JJ, Huisman HJ (2016). Visibility of prostate cancer on transrectal ultrasound during fusion with multiparametric magnetic resonance imaging for biopsy. Clin Imaging.

[10] Lee KS, Koo KC, Chung BH (2018). Quantitation of hypoechoic lesions for the prediction and Gleason grading of prostate cancer: a prospective study. World J Urol.

[11] Anderson M, Motta R, Chandrasekar S, Stokes M: Proposal for a standard default color space for the internet—srgb. In: Color and imaging conference: 1996: Society for Imaging Science and Technology; 1996: 238–245.

[12] Barrett T, Turkbey B, Choyke PL (2015). PI-RADS version 2: what you need to know. Clin Radiol.

[13] Meng X, Rosenkrantz AB, Mendhiratta N, Fenstermaker M, Huang R, Wysock JS, Bjurlin MA, Marshall S, Deng F-M, Zhou M (2016). Relationship between prebiopsy multiparametric magnetic resonance imaging (MRI), biopsy indication, and MRI-ultrasound fusion–targeted prostate biopsy outcomes. Eur Urol.

[14] Choi MH, Kim CK, Lee YJ, Jung SE (2019). Prebiopsy biparametric MRI for clinically significant prostate cancer detection with PI-RADS version 2: a multicenter study. AJR Am J Roentgenol.

[15] Wang R, Wang J, Gao G, Hu J, Jiang Y, Zhao Z, Zhang X, Zhang Y-D, Wang X (2017). Prebiopsy mp-MRI can help to improve the predictive performance in prostate cancer: a prospective study in 1,478 consecutive patients. Clin Cancer Res.

[16] Ouzzane A, Puech P, Lemaitre L, Leroy X, Nevoux P, Betrouni N, Haber G-P, Villers A (2011). Combined multiparametric MRI and targeted biopsies improve anterior prostate cancer detection, staging, and grading. Urology.

[17] Junqueira VCN, Zogbi O, Cologna A, Dos Reis RB, Tucci S, Reis LO, Westphalen AC, Muglia VF (2012). Is a visible (hypoechoic) lesion at biopsy an independent predictor of prostate cancer outcome?. Ultrasound Med Biol.

[18] Mottet N, van den Bergh RC, Briers E, Van den Broeck T, Cumberbatch MG, De Santis M, Fanti S, Fossati N, Gandaglia G, Gillessen S (2020). EAU-EANM-ESTRO-ESUR-SIOG guidelines on prostate cancer—2020 update. Part 1: screening, diagnosis, and local treatment with curative intent. Eur Urol.

[19] Rifkin M, McGlynn E, Choi H (1989). Echogenicity of prostate cancer correlated with histologic grade and stromal fibrosis: endorectal US studies. Radiology.

[20] Schoots IG (2018). MRI in early prostate cancer detection: how to manage indeterminate or equivocal PI-RADS 3 lesions?. Transl Androl Urol.

[21] Hermie I, Van Besien J, De Visschere P, Lumen N, Decaestecker K (2019). Which clinical and radiological characteristics can predict clinically significant prostate cancer in PI-RADS 3 lesions? A retrospective study in a high-volume academic center. Eur J Radiol.

[22] Hansen N, Koo B, Warren A, Kastner C, Barrett T (2017). Sub-differentiating equivocal PI-RADS-3 lesions in multiparametric magnetic resonance imaging of the prostate to improve cancer detection. Eur J Radiol.

[23] Al Awamlh BAH, Marks LS, Sonn GA, Natarajan S, Fan RE, Gross MD, Mauer E, Banerjee S, Hectors S, Carlsson S. Multicenter analysis of clinical and MRI characteristics associated with detecting clinically significant prostate cancer in PI-RADS (v2. 0) category 3 lesions. In: Urologic Oncology: Seminars and Original Investigations: 2020: Elsevier; 2020. p. e639–637. e615. 10.1016/j.urolonc.2020.03.019 PMC7328785 32307327

[24] Yang S, Zhao W, Tan S, Zhang Y, Wei C, Chen T, Shen J (2020). Combining clinical and MRI data to manage PI-RADS 3 lesions and reduce excessive biopsy. Transl Androl Urol.

